# High expression of TTC21A predicts unfavorable prognosis and immune infiltrates in clear cell renal cell carcinoma

**DOI:** 10.3389/fgene.2022.967378

**Published:** 2022-11-03

**Authors:** Junhao Lin, DeYong Nong, Wei Wang, Xiaobin Guo, ChunLin Li, BingCai Li, Haojian Wang, Zhi Chen, XiMing Li, GuiHai Huang, Wei Li

**Affiliations:** ^1^ Department of Urology, The First Clinical Medical College of Jinan University, Jinan University, Guangzhou, China; ^2^ Department of Urology, The People’s Hospital of Guangxi Zhuang Autonomous Region, Nanning, China

**Keywords:** tetratricopeptide repeat domain 21A, prognosis, immune infiltrating, clear cell renal cell carcinoma, bioinformactics analysis

## Abstract

**Background**: Clear cell renal cell carcinoma (ccRCC) is the most common pathological type of renal cell carcinoma. Tetratricopeptide repeat domain 21A (TTC21A), known as a component of intraflagellar transport complex A which is essential for the function of cilia, However, the role of TTC21A remains unclear in ccRCC. For the first time, we explore the role and potential mechanism of TTC21A in ccRCC based on multiple databases.

**Methods**: TTC21A expression across all TCGA tumor was analyzed *via* Tumor Immune Estimation Resource (TIMER) site. The correlation between TTC21A and clinicopathologic characteristics of ccRCC was analyzed with TCGA database. The diagnostic and prognostic value of TTC21A was evaluated by receiver operation characteristic curve, Kaplan-Meier plotter and Cox regression respectively. Moreover, functional enrichment analysis of TTC21A and the co-expression genes were performed by Gene Set Enrichment Analysis. The correlation of TTC21A and immune infiltration were evaluated by single sample Gene Set Enrichment Analysis.

**Results**: Pan-cancer analysis indicated that TTC21A was highly expressed in ccRCC and other cancer. In addition, elevated expression of TTC21A was associated with worse overall survival in ccRCC patients. Functional enrichment analysis showed that TTC21A and the co-expressed genes enriched in glucose metabolism and energy metabolism. Moreover, TTC21A expression was associated with infiltrating levels of dendritic cell, nature killer cell and other immune marker sets.

**Conclusion**: The results of analysis indicate that expression of TTC21A is associated with poor prognosis and immune infiltrating in ccRCC, which suggested TTC21A might be used as a potential predictor and target of treatment in ccRCC.

## Introduction

Renal cell carcinoma (RCC) ranked the third most common urologic cancers worldwide (Sung et al., 2021). Furthermore, it represents the rising and the first mortality rate in the common urologic cancers (Partin et al., 2015). Despite advances in surgery for localized RCC, the efficacy of treatment for advanced or metastatic RCC is inadequate (Barata and Rini, 2017). Anatomical factors, including distant metastasis and perinephric invasion, are the important prognostic factors of RCC (Ljungberg et al., 2015). Therefore, lots of researches focused on advanced or metastatic RCC. Previous study had reported that immune infiltration of tumor was correlated with prognosis in RCC (Zhang et al., 2019). Tumor-infiltration immune cells, including CD4^+^ T cells, CD8^+^ T cells and Tfh cells, could regulate cancer progression in RCC, which could be regarded as targets for novel drugs to improve outcome (Vesely et al., 2011; Wang et al., 2018).

Renal cell carcinoma is consisted of three primary histologic subtypes: clear cell RCC, papillary RCC (pRCC) and chromophobe RCC (chRCC). Clear cell renal cell carcinoma (ccRCC or KIRC) is the most common subtype of RCC, which account for 70–80% of all RCC (Partin et al., 2015). Compared to pRCC or chRCC, the prognosis of ccRCC is worse, and 5-year relative survival of metastatic ccRCC is less than 10% (Cohen and McGovern, 2005). With the novel immunotherapy, the patients with ccRCC could have a longer overall survival. Nowadays, immune checkpoint inhibitor has been recommended as a treatment for advanced or metastatic RCC. Programed death-1 (PD-1) and cytotoxic T lymphocyte associated antigen 4 (CTLA4) has been considered as second-line therapy for RCC (Ljungberg et al., 2015).

Tetratricopeptide repeat domain 21A (TTC21A), a gene with protein coding, is a component of intraflagellar transport complex A (IFT-A complex) which is essential for the function of cilia (Picariello et al., 2019). Some studies had indicated that cilia contributed to tumorigenesis. Downregulation of cilia expression could be observed in renal cell carcinoma, pancreatic cancer, cholangiocarcinoma, and melanoma (Schraml et al., 2009; Kim et al., 2011; Gradilone et al., 2013). Besides, there were some researches indicated that IFT complex was associated with different types of cancers (Pidugu et al., 2019; Jiang et al., 2021; Tan et al., 2021). However, the investigations of TTC21A in tumor progression were limited. Previous studies reported TTC21A was associated with the prognosis and immune infiltrating level in lung adenocarcinoma (LUAD) and colorectal cancer (Wang et al., 2020; Li et al., 2022). The purpose of present study is to explore the role of TTC21A in clear cell renal cell carcinoma.

The data of clear cell renal cell carcinoma was downloaded from The Cancer Genome Atlas (TCGA) database. The diagnostic capacity of TTC21A expression in ccRCC was evaluated by receiver operation characteristic (ROC) curve. Meanwhile, the influence of TTC21A on the prognosis of ccRCC was analyzed by Kaplan-Meier plotter and Cox regression. Furthermore, the single sample Gene Set Enrichment Analysis (ssGSEA) was used to examine the correlations between TTC21A and different kinds of tumor-infiltrating immune cells (TILCs) in ccRCC. The results of present study could improve the understanding of the role of TTC21A in ccRCC, which make it possible to become a novel biomarker to predict tumor prognosis and immune infiltration for ccRCC patients.

## Materials and methods

### TCGA datasets analysis

Transcriptional expression data of TTC21A in clear cell renal cell carcinoma was downloaded from The Cancer Genome Atlas (TCGA, https://portal.gdc.cancer.gov) database, which included 539 ccRCC tissues and 72 normal tissues. The gene expression with FPKM type was converted into the type of TPM for further analysis. The result was visualize using GraphPad Prism software (version 6.0; GraphPad software, Inc.). Statistical software R (https://www.r-project.org; version 3.6.3) was used for further analysis of TCGA database. Transcript data and clinical data of 33 TCGA cancer types was also downloaded from TCGA database for univariate cox regression analysis, and the result was visualized with a forest plot by using the “forestplot” package of R (https://CRAN.R-project.org/package=forestplot; version 2.0.1). The discrimination ability of TTC21A in KIRC was evaluated by ROC curve, and ROC curve was performed by “pROC” package of R (https://CRAN.R-project.org/package=pROC; version 1.18.0) (Robin et al., 2011). Multivariate analysis of overall survival was achieved by “survival” package of R (https://CRAN.R-project.org/package=survival; version 3.2–13), and nomogram and calibration plot were constructed by “survival” package and “rms” package of R (https://CRAN.R-project.org/package=rms; version 6.2–0) (Iasonos et al., 2008).

### Kaplan-meier plotter database analysis

Kaplan-Meier Plotter Database can be used to analyze the effect of genes on survival in different types of cancer (Nagy et al., 2021). The correlation between TTC21A expression and overall survival in ccRCC was analyzed by Kaplan-Meier plotter (http://www.kmplot.com/analysis/). The log-rank *p* value and the hazard ratio were calculated.

### LinkedOmics database analysis

The LinkedOmics database (http://www.linkedomics.org/login.php) is a publicly available platform for analyzing multi-omics data of 32 TCGA cancer types (Vasaikar et al., 2018). TTC21A co-expression was analyzed by using Pearson’s test, and the results were presented by volcano plot and heatmap. Gene Ontology (GO) term enrichment analysis and Kyoto Encyclopedia of Genes and Genomes (KEGG) pathway enrichment analysis of TTC21A co-expression genes was performed by using gene set enrichment analysis (GSEA).

### Gene set enrichment analysis

Gene Set Enrichment Analysis (GSEA) was conducted with a ccRCC cohort from TCGA database by using the GSEA software (www.gsea-msigdb.org/gsea/index.jsp) (Mootha et al., 2003; Subramanian et al., 2005). GSEA was performed for Gene Ontology (GO) term enrichment and Kyoto Encyclopedia of Genes and Genomes (KEGG) pathway enrichment with 1000 permutations, and corresponding gene sets (C5. go. v7.5. symbols. gmt and C2. cp. kegg. v7.5. symbols. gmt) were used as reference gene sets (Liberzon et al., 2011; Liberzon et al., 2015). False discovery rate (FDR) q-value < 0.25, |normalized enrichment score (NES)| > 1 and adjust *p*-value < 0.05 were identified as significant.

### TIMER database analysis

TIMER is a comprehensive web for analysis of immune infiltrates in different cancer types (http://timer.comp-genomics.org/) (Li et al., 2020). The TIMER database comprises 10897 samples from The Cancer Genome Atlas (TCGA). The expression of TTC21A in different cancer types was analyzed by TIMER database.

### Immune infiltration analysis

The correlation between TTC21A and tumor-infiltrating immune cells was analyzed by ssGSEA with “GSVA” package of Bioconductor (https://bioconductor.org/packages/GSVA/) (Hänzelmann et al., 2013). The enrichment score of immunocyte was quantified based on the markers of 24 types immunocyte as reported previously (Bindea et al., 2013). The correlation between TTC21A and immune infiltrates cells was analyzed by Spearman correlation.

### Statistical analysis

The TTC21A expression between tumor and normal tissues was analyzed with Wilcoxon rank sum test. The correlation between TTC21A expression and clinicopathological characteristics were compared with Pearson χ^2^ test and Wilcoxon rank sum test. Survival curves were generated in Kaplan-Meier Plotter Database, and were displayed hazard ratio (HR) and *p* - values. The correlation between TTC21A expression and immune infiltration were analyzed by Spearman correlation. *p* < 0.05 was considered significant.

## Results

### The expression of TTC21A in different types of cancers

**FIGURE 1:**
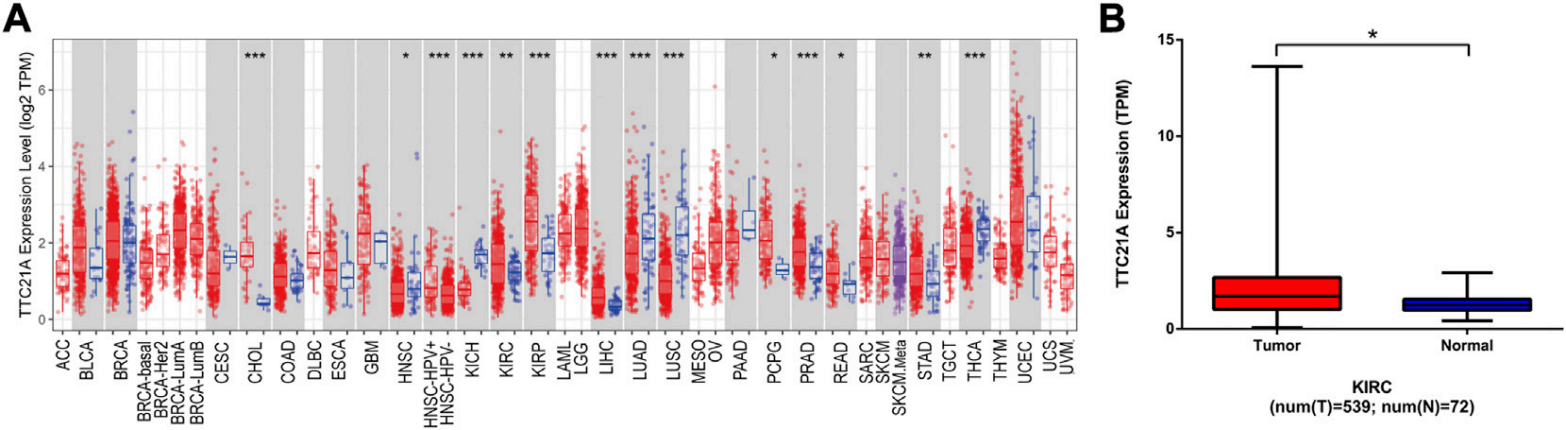
Differential expression of TTC21A. **(A)** Differential expression of TTC21A in 33 types cancers (*p < 0.05, **p < 0.01, ***p < 0.001) **(B)** Differential expression of TTC21A in ccRCC and normal tissues from TCGA database (*p < 0.05).

The expression of TTC21A in different types of cancers was evaluated by using TERM database. The TTC21A expression between tumor and normal tissues in TCGA tumors was shown in Figure 1A. The expression of TTC21A was significantly higher in CHOL (Cholangiocarcinoma), KIRC (Kidney Renal Clear Cell Carcinoma), KIRP (Kidney Renal Papillary Cell Carcinoma), LIHC (Liver Hepatocellular Carcinoma), PCPG (Pheochromocytoma and Paraganglioma), PRAD (Prostate Adenocarcinoma), READ (Rectum Adenocarcinoma), STAD (Stomach Adenocarcinoma) compared with that of normal tissues. However, TTC21A expression was significantly lower in HNSC (Head and Neck Cancer), KICH (Kidney Chromophobe), LUAD (Lung Adenocarcinoma), LUSC (Lung Squamous Cell Carcinoma), THCA (Thyroid Carcinoma) compared with normal tissues. Analysis of ccRCC cohort in TCGA database revealed that TTC21A expression was significantly elevated in tumor tissues compared with adjacent normal tissues (Figure 1B).

### Correlation between TTC21A expression and clinicopathologic characteristics

**FIGURE 2:**
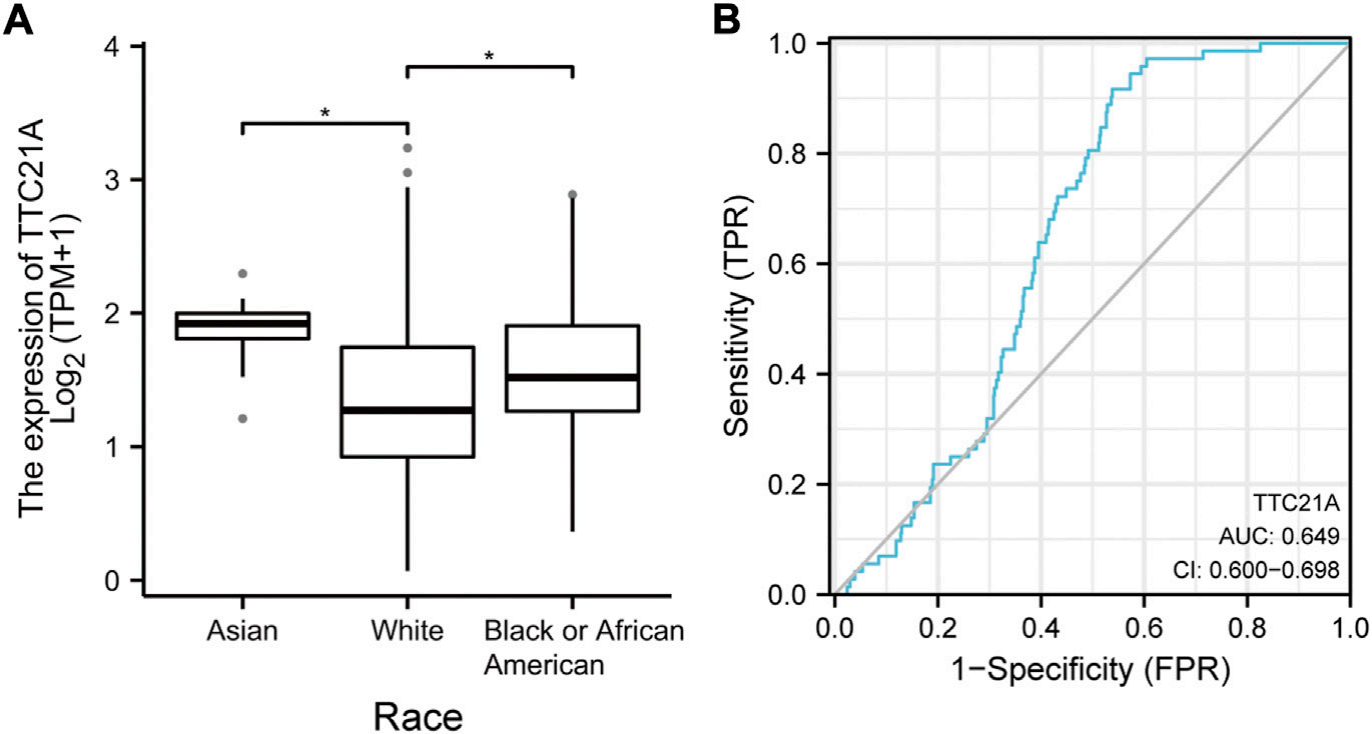
Correlation of TTC21A and clinicopathologic characteristics in ccRCC. **(A)** TTC21A expression was associated with different races (*p < 0.05) **(B)** Receiver operation characteristic (ROC) curve of TTC21A (TPR true positive rate, FPR false positive rate).

539 KIRC samples were classified into two groups by the median value of TTC21A expression. As shown in Table 1, high expression of TTC21A was related with race (*p* = 0.001). Whereas, the other clinicopathologic characteristics had no different between high- and low-expression groups. Different races showed significant difference in the expression of TTC21A (Figure 2A). Besides, the area under the ROC curve (AUC) showed a diagnostic accuracy of TTC21A in KIRC, however, the accuracy was low (AUC = 0.649, 95%CI = 0.600–0.698) (Figure 2B).

**TABLE 1:** clinicopathologic characteristics between high- and low-expression of TTC21A in ccRCC.

Characteristic	Low expression of TTC21A	High expression of TTC21A	p
n	269	270	
T stage, n (%)			0.544
T1	133 (24.7%)	145 (26.9%)	
T2	37 (6.9%)	34 (6.3%)	
T3	95 (17.6%)	84 (15.6%)	
T4	4 (0.7%)	7 (1.3%)	
N stage, n (%)			0.735
N0	117 (45.5%)	124 (48.2%)	
N1	9 (3.5%)	7 (2.7%)	
M stage, n (%)			1.000
M0	223 (44.1%)	205 (40.5%)	
M1	41 (8.1%)	37 (7.3%)	
Pathologic stage, n (%)			0.661
Stage I	129 (24.1%)	143 (26.7%)	
Stage II	30 (5.6%)	29 (5.4%)	
Stage III	66 (12.3%)	57 (10.6%)	
Stage IV	43 (8%)	39 (7.3%)	
Gender, n (%)			0.131
Female	84 (15.6%)	102 (18.9%)	
Male	185 (34.3%)	168 (31.2%)	
Race, n (%)			0.001
Asian	1 (0.2%)	7 (1.3%)	
Black or African American	18 (3.4%)	39 (7.3%)	
White	247 (46.4%)	220 (41.4%)	
Histologic grade, n (%)			0.111
G1	4 (0.8%)	10 (1.9%)	
G2	109 (20.5%)	126 (23.7%)	
G3	105 (19.8%)	102 (19.2%)	
G4	44 (8.3%)	31 (5.8%)	
Age, mean ± SD	60.82 ± 12.27	60.44 ± 11.93	0.715

### TTC21A expression is correlated with survival in ccRCC

**FIGURE 3:**
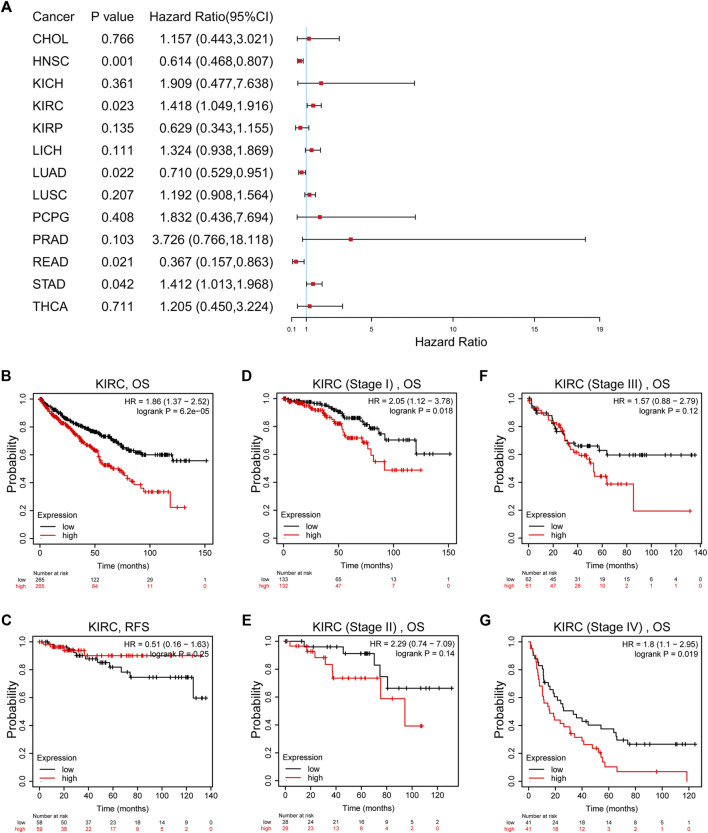
Survival analysis of TTC21A in ccRCC. **(A)** Correlation between TTC21A expression and overall survival of different types cancers. **(B)** Overall survival was significantly higher in patients with low TTC21 expression (HR = 1.86, 95%CI = 1.37 to 2.52, log-rank test, *p* < 0.05) **(C)** Relapse free survival was not significantly higher in patients with low TTC21 expression (HR = 0.51, 95%CI = 0.16 to 1.63, log-rank test, *p* > 0.05). **(D–G)** Overall survival in stage I and stage IV of ccRCC were significantly higher in patients with low TTC21 expression, while overall survival in stage II and stage III were not significant.

Univariate Cox regression indicated that high TTC21A expression was associated with overall survival in KIRC, HNSC, LUAD, READ and STAD (Figure 3A). Kaplan-Meier survival curve revealed that high expression of TTC21A was associated with worse prognosis in KIRC (OS HR = 1.86, 95%CI = 1.37 to 2.52, log-rank test, *p* < 0.05) (Figure 3B). In addition, the elevated TTC21A expression led to significantly shorter overall survival (OS) in different stage of ccRCC (Stage I HR = 2.05, 95%CI = 1.12 to 3.78, log-rank test, *p* < 0.05; Stage IV HR = 1.80, 95%CI = 1.10 to 2.95, log-rank test, *p* < 0.05) (Figures 3D,G). However, High TTC21A expression was not associated with relapse free survival (RFS) and stage II, Stage III disease in patients with ccRCC (Figures 3C,E,F).

**FIGURE 4:**
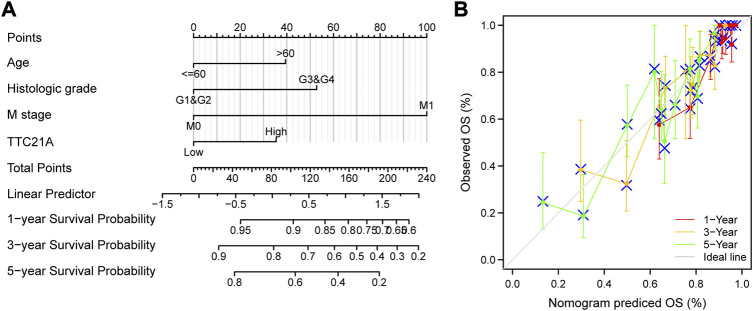
Predictive ability of TTC21A in ccRCC. **(A)** Nomogram for predicting overall survival of ccRCC. **(B)** Calibration curve of the nomogram.

Univariate and multivariate Cox analyses indicated that M stage (*p* < 0.001), age (*p* < 0.05), histologic grade (*p* < 0.05) and TTC21A expression (*p* < 0.05) were independently associated with overall survival (Table 2). Based on multivariate Cox regression, the nomogram was conducted (Figure 4A). Potential covariates included M stage, age, histologic grade and TTC21A expression. The concordance index (C-index) was used to evaluate the predictive ability of the prognostic model (C-index = 0.741, 95%CI = 0.722–0.760). Calibration curve of the nomogram was made, and the bias-corrected lines were approaching the ideal line (Figure 4B).

**TABLE 2:** Univariate and multivariate Cox analyses of OS in ccRCC patients.

Characteristics	Total(N)	Univariate analysis		Multivariate analysis	
		Hazard ratio (95% CI)	p Value	Hazard ratio (95% CI)	p Value
T stage	539				
T1&T2	349	References			
T3&T4	190	3.228 (2.382–4.374)	<0.001	1.450 (0.637–3.301)	0.375
N stage	257				
N0	241	References			
N1	16	3.453 (1.832–6.508)	<0.001	1.483 (0.731–3.010)	0.275
M stage	506				
M0	428	References			
M1	78	4.389 (3.212–5.999)	<0.001	2.537 (1.488–4.327)	<0.001
Gender	539				
Male	353	References			
Female	186	1.075 (0.788–1.465)	0.648		
Age	539				
≤60	269	References			
>60	270	1.765 (1.298–2.398)	<0.001	1.653 (1.077–2.538)	0.022
TTC21A	539				
Low	269	References			
High	270	1.418 (1.049–1.916)	0.023	1.756 (1.129–2.733)	0.013
Pathologic stage	536				
Stage I&Stage II	331	References			
Stage III&Stage IV	205	3.946 (2.872–5.423)	<0.001	1.556 (0.602–4.018)	0.361
Histologic grade	531				
G1&G2	249	References			
G3&G4	282	2.702 (1.918–3.807)	<0.001	1.685 (1.020–2.781)	0.041
Race	532				
Asian	8	References			
White&Black or African American	524	1.812 (0.253–12.963)	0.554		

### Biological functions of TTC21A in ccRCC

**FIGURE 5:**
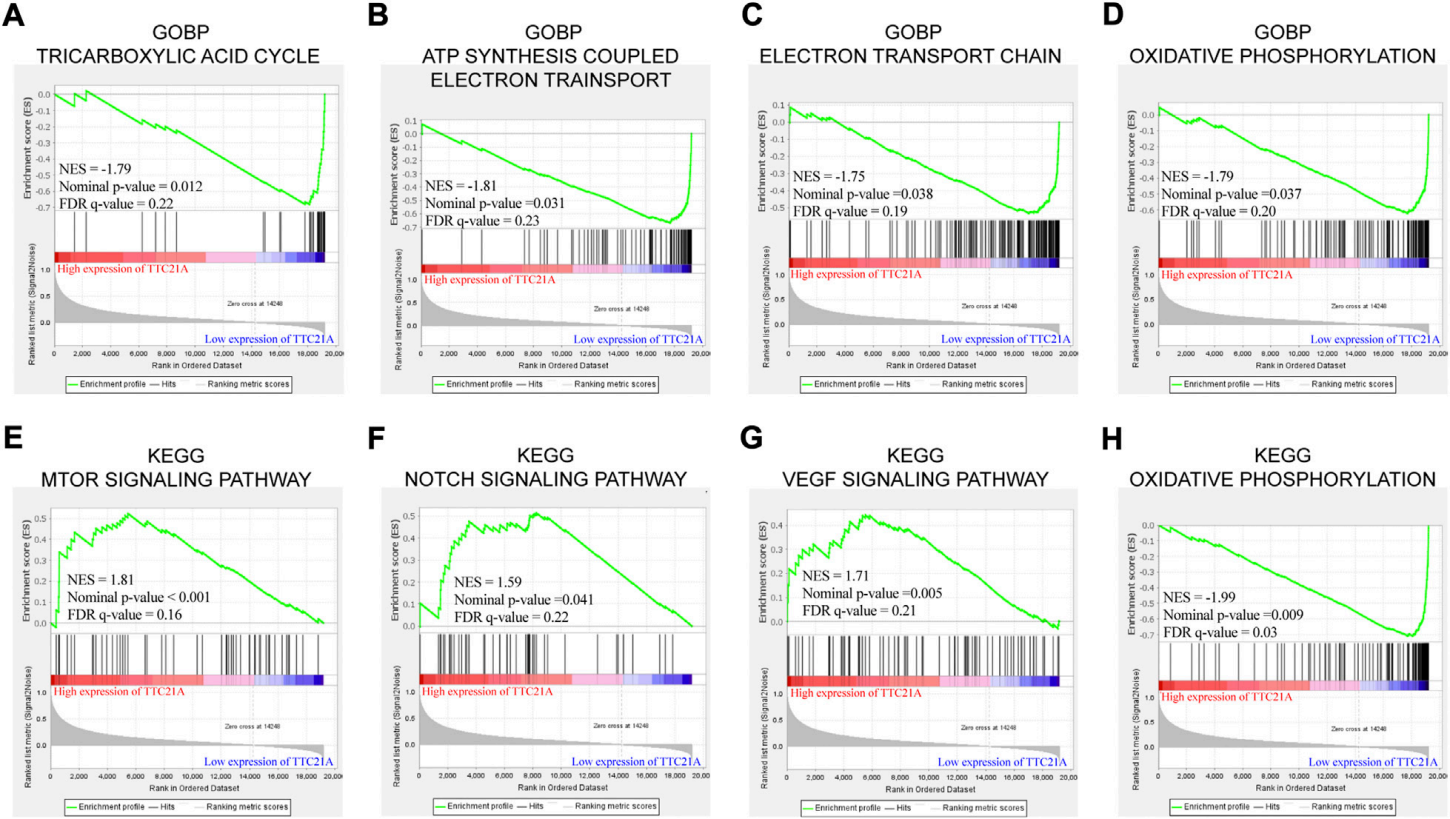
Enrichment analysis of TTC21A in ccRCC. Tricarboxylic acid cycle **(A)**, ATP synthesis coupled electron transport **(B)**, electron transport chain **(C)** and oxidative phosphorylation **(D)** were enriched in biological process of ccRCC. MTOR signaling pathway **(E)**, NOTCH signaling pathway **(F)**, VEGF signaling pathway **(G)** and oxidative phosphorylation **(H)** were enriched in KEGG pathway.

GO term enrichment analysis and KEGG pathway enrichment analysis between low and high TTC21A expression were performed by GSEA. GO term enrichment analysis in biological process (BP) revealed that high expression of TTC21A was associated with ‘ATP synthesis coupled electron transport’, “electron transport chain”, oxidative phosphorylation’ and “tricarboxylic acid cycle” (Figures 5A–D). KEGG pathway enrichment analysis revealed that high expression of TTC21A was associated with signaling pathway of mTOR, Notch, VEGF and oxidative phosphorylation (Figures 5E–H).

### The co-expression networks of TTC21A in ccRCC

**FIGURE 6:**
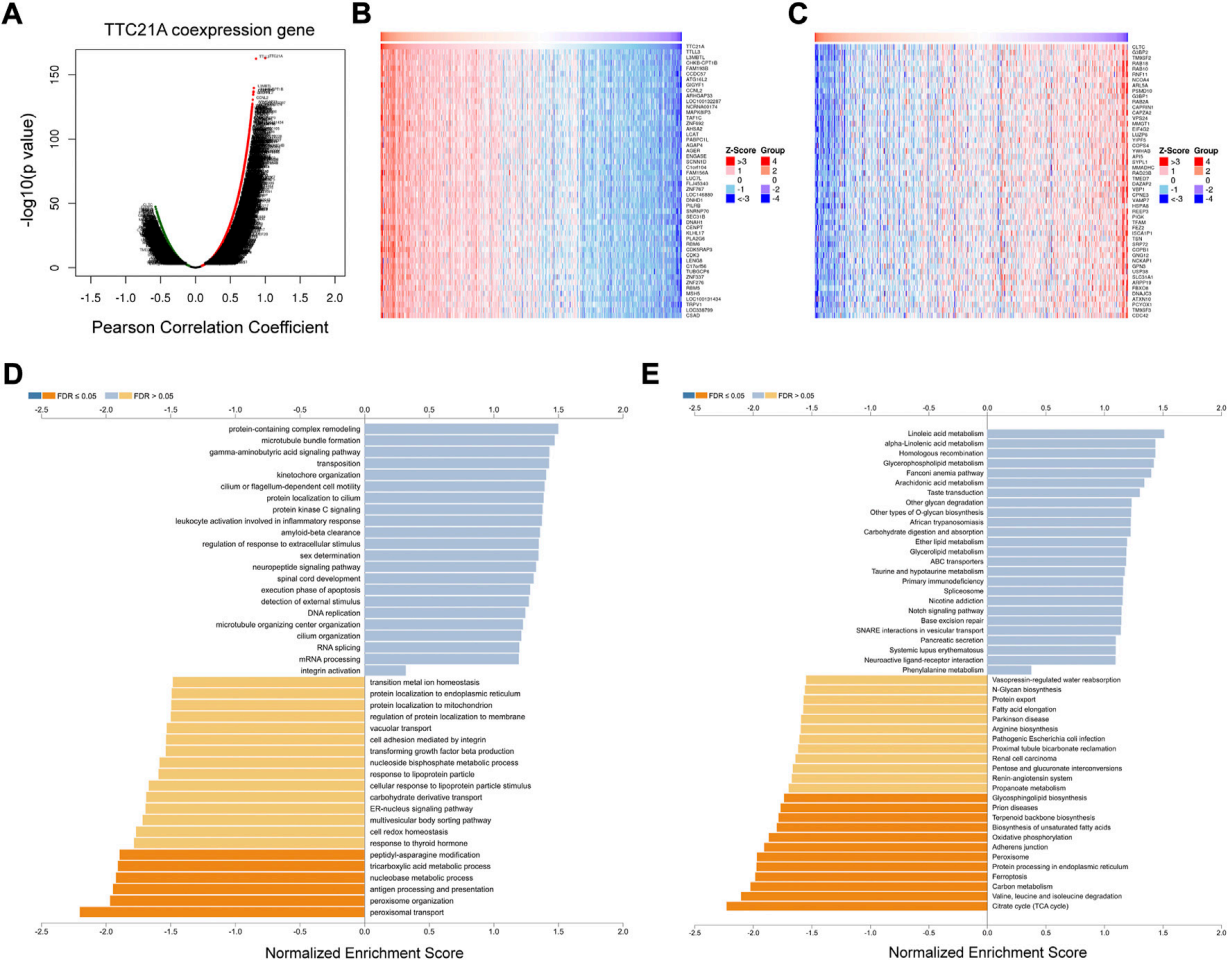
Analysis of the co-expression genes of TTC21A in ccRCC. **(A)** The co-expression genes of TTC21A in ccRCC. **(B)** Heat map of top 50 genes positively correlated with TTC21A **(C)** Heat map of top 50 genes negatively correlated with TTC21A. **(D)** Enrichment analysis of the co-expression genes in biological process of ccRCC. **(E)** Enrichment analysis of the co-expression genes in KEGG pathway of ccRCC.

The database of LinkedOmics was performed to evaluate the co-expression networks of TTC21A in ccRCC. 8463 genes (red dots) were positively correlated with TTC21A significantly, whereas 4634 genes (green dots) were negatively correlated with TTC21A (*p* < 0.05) (Figure 6A). The top 50 genes positively or negatively correlated with TTC21A were presented in the heatmap respectively (Figures 6B,C). Furthermore, GO term enrichment analysis of TTC21A co-expression genes was performed by gene set enrichment analysis (GSEA). The results of GO enrichment analysis in biological processes (BP) indicated that the activities of peroxisome organization, peptidyl-asparagine modification, nucleobase metabolic process, tricarboxylic acid metabolic process, and peroxisomal transport were inhibited by co-expression genes (Figure 6D). KEGG pathway enrichment suggested that TTC21A co-expression genes were negatively associated with glycosphingolipid biosynthesis, prion diseases, terpenoid backbone biosynthesis, biosynthesis of unsaturated fatty acids, adherens junction, ferroptosis, protein processing in endoplasmic reticulum, oxidative phosphorylation, peroxisome, carbon metabolism, valine, leucine and isoleucine degradation, and citrate cycle (TCA cycle) (Figure 6E).

### Correlation analysis between TTC21A and immune infiltration in ccRCC

**FIGURE 7:**
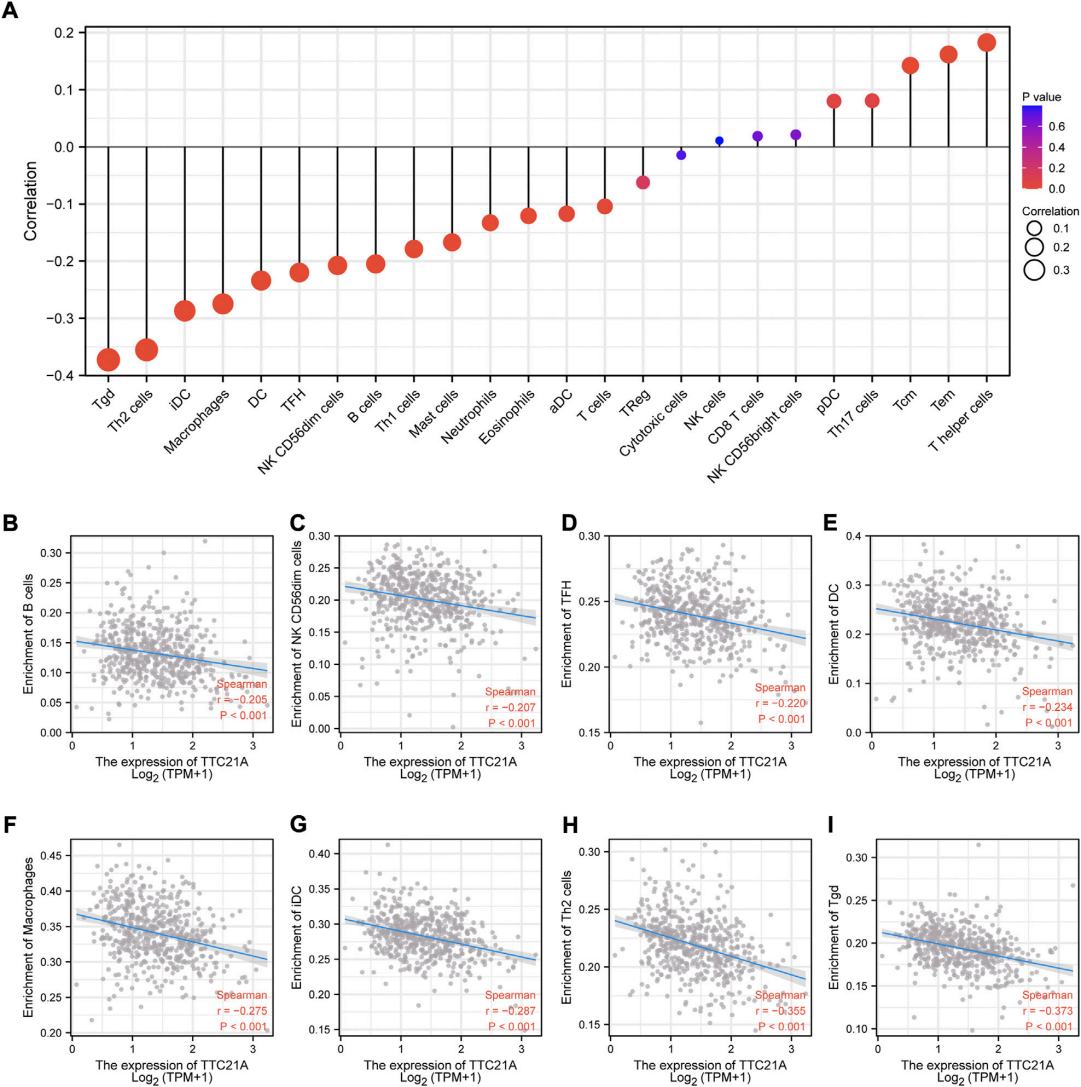
Association of TTC21A with immune infiltration. **(A)** Association of TTC21A with immune infiltration (Tem T effector memory, Tcm T central memory, Th helper T cells, pDC plasmacytoid DC, NK natural killer, Treg regulatory T cells, aDC activated DC, TFH T follicular helper, DC dendritic cells, iDC immature DC, Tgd T gamma delta). **(B–I)** TTC21A was negatively correlated with infiltration of B cells, NK CD56dim cells, TFH, DC, macrophages, iDC, Th2 cells and Tgd.

We investigated the correlation between TTC21A and tumor-infiltrating immune cells in ccRCC. The results of analysis indicated that TTC21A correlated with most tumor-infiltrating immune cells in ccRCC (Figure 7A). In addition, TTC21A was negatively correlated with B cells, NK CD56dim cells, T follicular helper (TFH), dendritic cells (DC), macrophages, immature dendritic cells (iDC), Type 2 T helper (Th2) cells and T gamma delta (Tgd) (Figures 7B–I).

## Discussion

TTC21A is a protein-coding gene associated with male infertility, and might involve in reduced motility of spermatozoa and multiple morphological abnormalities of the sperm flagella (MMAF) (Liu et al., 2019). In addition, a recent study reported high expression of TTC21A predicts favorable prognosis in lung adenocarcinoma (Wang et al., 2020). In our study, we found that TTC21A was over-expressed in ccRCC, which correlated with poor prognosis. Furthermore, our analysis indicated that immune infiltration levels were correlated with the expression of TTC21A in ccRCC. Thus, TTC21A might be a potential biomarker for ccRCC.

In our study, the expression of TTC21A was calculated in different types of cancer. The result revealed that TTC21A was highly expressed in ccRCC tissues compared to normal tissues. The analysis of clinicopathologic features showed that TTC21A expression was correlated with race. Whereas, other clinicopathologic features were not correlated with high expression of TTC21A. In addition, the ROC analysis suggested that TTC21A might be a diagnostic marker for ccRCC, although the diagnostic power was relatively low. The prognostic value of TTC21A in ccRCC was also evaluated based on the Kaplan-Meier survival analysis and multivariate Cox analysis, which showed that TTC21A was an independent risk factor for ccRCC, and high TTC21A expression was associated with poor OS. All these results suggested that TTC21A might be a potential prognostic marker of ccRCC.

Gene set enrichment analysis and co-expression analysis were performed to explore the possible mechanism of TTC21A in ccRCC. The enrichment analysis of biological process revealed that TTC21A was involved in tricarboxylic acid cycle (TCA cycle), oxidative phosphorylation (OxPhos) and electron transport in ccRCC, which were related to glucose and energy homeostasis. Otherwise, the enrichment analysis of pathway showed that TTC21A was associated with mTOR, Notch, VEGF signaling pathway and oxidative phosphorylation. Glucose homeostasis disorder is an essential part of the Warburg effect, and it was widely accepted the Warburg effect contributes to tumorigenesis, proliferation, angiogenesis, metastasis and immune evasion in tumor (Vaupel et al., 2019). The Warburg effect is mainly characterized by enhanced aerobic glycolysis and mitochondrial dysfunction. Mitochondrial dysfunction can lead to inhibition of OxPhos and TCA cycle *via* mutation of mitochondrial enzymes (King et al., 2006; Ratcliffe, 2007; Rasheed and Tarjan, 2018; Schmidt et al., 2020; Vaupel and Multhoff, 2021). Research on the mechanism reveal that the Warburg effect is triggered by activation of hypoxia-inducible factor-1 (HIF-1), oncogene activation (e.g. K-Ras, Akt, mTORC1), altered signaling pathways (e.g. PI3K-Akt-mTORC1 signaling pathway and Ras-Raf-MEK-ERK-cMyc signaling pathway) and inactivation of tumor suppressors (e.g. p53 and PTEN) (Senyilmaz and Teleman, 2015; Srinivasan et al., 2016; Cassim et al., 2020; Grasso et al., 2020). Based on the results of enrichment analysis, we hypothesized TTC21A may affect progression of ccRCC by regulating glucose homeostasis *via* mTOR pathway. However, the hypothesis needs further experiments *in vivo* for validation.

It is well appreciated that immune infiltration is critical for the progression of diverse tumors (Gajewski et al., 2013). Clear cell renal cell carcinoma is characterized with enrich immune cell infiltrate, including CD4^+^ cells, CD8^+^ cells and nature killer (NK) cells (Kopecký et al., 2007; Komohara et al., 2011). Accumulated evidence shown metabolic reprogramming was correlated with tumor microenvironments (TMEs) (Vaupel, 2004; Vaupel and Multhoff, 2018). Metabolic disorder in tumor can shape tumor microenvironments, and lead to immunosuppression and immune evasion (Li and Simon, 2020). Taken together, TTC21A was related to some metabolic processes, which arise the possibility that TTC21A may be associated with immune infiltration. The analysis of the interrelation between TTC21A expression and diverse immune infiltration cell in ccRCC showed the expression of TTC21A was negatively correlated with infiltration level of B cells, NK CD56dim cells, TFH, DC, macrophages, iDC, Th2 cells and Tgd. Accumulated lactate produced by aerobic glycolysis could inhibit NK cells, T cells and M1 state macrophages. In addition, lactate could prohibit the presentation of tumor antigens from DCs to other immune cells (Pavlova and Thompson, 2016). Based on our results, we hypothesized that immune infiltration correlated with TTC21A may contribute to the poor prognosis of ccRCC.

In conclusion, high expression of TTC21A is associated with poor prognosis in ccRCC. Moreover, the metabolic reprogramming and immune infiltration may be the possible mechanism of TTC21A. Obviously, all of the conclusions still need well designed experiments to further confirm. Another limitation was the clinical information was not comprehensive in TCGA database. The significance of present study was to provide a novel predictor and target for local advanced or metastatic RCC.

## Data Availability

The original contributions presented in the study are included in the article/supplementary material, further inquiries can be directed to the corresponding author.
